# Antagonistic Peptides That Specifically Bind to the First and Second Extracellular Loops of CCR5 and Anti-IL-23p19 Antibody Reduce Airway Inflammation by Suppressing the IL-23/Th17 Signaling Pathway

**DOI:** 10.1155/2020/1719467

**Published:** 2020-04-28

**Authors:** Yingli Zhang, Rongrong Liang, Aicen Xie, Wenqian Shi, Huarong Huang, Yingqiang Zhong

**Affiliations:** ^1^Department of Pediatrics, Sun Yat-sen Memorial Hospital, Sun Yat-sen University, Guangzhou, Guangdong 510120, China; ^2^Department of Gastroenterology, Sun Yat-sen Memorial Hospital, Sun Yat-sen University, Guangzhou, Guangdong 510120, China

## Abstract

Asthma is a heterogeneous chronic inflammatory disorder of the airways with a complex etiology, which involves a variety of cells and cellular components. Therefore, the aim of the study was to investigate the effects and mechanisms of antagonistic peptides that specifically bind to the first and second extracellular loops of CCR5 (GH and HY peptides, respectively) and anti-interleukin-23 subunit p19 (anti-IL-23p19) in the airway and thereby mediate inflammation and the IL-23/T helper 17 (Th17) cell pathway in asthmatic mice. An experimental asthma model using BALB/c mice was induced by ovalbumin (OVA) and treated with peptides that are antagonistic to CCR5 or with anti-IL-23p19. The extents of the asthmatic inflammation and mucus production were assessed. In addition, bronchoalveolar lavage fluid (BALF) was collected, the cells were counted, and the IL-4 level was detected by ELISA. The IL-23/Th17 pathway-related protein and mRNA levels in the lung tissues were measured, and the positive production rates of Th17 cells in the thymus, spleen, and peripheral blood were detected. The groups treated with one of the two peptides and/or anti-IL-23p19 showed significant reductions in allergic inflammation and mucus secretion; decreased expression levels of IL-23p19, IL-23R, IL-17A and lactoferrin (LTF); and reduced proportions of Th17 cells in the thymus, spleen, and peripheral blood. Specifically, among the four treatment groups, the anti-IL-23p19 with HY peptide group exhibited the lowest positive production rate of Th17 cells. Our data also showed a significant and positive correlation between CCR5 and IL-23p19 protein expression. These findings suggest that the administration of peptides antagonistic to CCR5 and/or anti-IL-23p19 can reduce airway inflammation in asthmatic mice, most likely through inhibition of the IL-23/Th17 signaling pathway, and the HY peptide can alleviate inflammation not only through the IL-23/Th17 pathway but also through other mechanisms that result in the regulation of inflammation.

## 1. Introduction

Asthma is a chronic inflammatory disease characterized by airway inflammation, mucus secretion, airway hyperresponsiveness (AHR), and airway remodeling [1, 2]. The results from a recent 10-year multicenter study showed that the incidence of severe or refractory asthma has been constantly increasing and is accompanied by poor prognosis despite years of standardized treatment [3]. Therefore, the identification of a therapeutic target with greater effectiveness would be of great clinical significance.

Previous studies have suggested that a Th1/Th2 imbalance is closely related to the development of asthma [4]. Interferon *γ* (IFN-*γ*) secreted by Th1 cells can inhibit eosinophils infiltration, mucus secretion, and other pathologic features that are mediated by antigen-specific Th2 cells and their cytokines [5]. However, increased numbers of neutrophils in the airway are observed in severe or refractory asthma [6]. The Th1/Th2 imbalance theory does not account for all mechanisms of asthma. Therefore, the discovery of the IL-23/Th17 signaling pathway complements the knowledge of the immunoregulatory mechanism of asthma, which shows correlation with a poor response to inhaled corticosteroids. IL-23 is primarily produced by dendritic cells (DCs), macrophages, lymphocytes, and endothelial cells; is critical to microbial exposure; and participates in the regulation of various inflammatory diseases [7]. Importantly, IL-23 not only induces the secretion of IL-17 from Th17 cells and the recruitment of neutrophils to the airways but also enhances Th2 cell responses and mediates the involvement of eosinophils in the inflammatory response of asthma [8]. A clinical study showed that the serum level of IL-23 in asthmatic patients is closely related to the degree of airflow limitation. In addition, the results of experiments using transbronchial biopsy samples from patients with severe asthma showed that Th17 cell cytokines, such as IL-17A, IL-17F, and IL-21, are expressed in the airway and that the higher levels of neutrophils were observed [9]. In particular, IL-23 consists of a p40 subunit of IL-12 and a p19 subunit specific to IL-23 [10]. A study using a murine asthma model showed that IL-23p19-deficient (IL-23p19^−/−^) mice exhibit lower airway resistance and a decreased number of inflammatory factors related to the IL-23/Th17 axis and decreased IL-23 production by DCs [11]. These findings indicate the importance of the IL-23/Th17 axis in the development of asthma.

Disposal of inflammation cytokines is a critical goal for inflammation disease therapeutics, and inhibiting inflammation cytokine secretion is a potential strategy to achieve this. CCR5 is an important chemokine receptor in innate and adaptive immunity and is primarily expressed by DCs, macrophages, and lymphocytes [12]. Several studies have shown that CCR5 binds to its ligands when induced by stimuli, and this receptor also plays an important role in the recruitment of immune cells to inflammatory sites and induces the secretion of cell mediators [13]. At present, CCR5 antagonists are being used to treat acquired immune deficiency syndrome (AIDS), rheumatoid arthritis, inflammatory bowel disease, and atherosclerosis [14]. A study of the OVA-induced asthma model in CCR5^−/−^ mice showed a reduction in airway hyperresponsiveness accompanied by decreases in the IL-4 and IL-13 levels and the number of leukocytes in the BALF [15]. Therefore, CCR5 is thought to be involved in the regulation of the airway inflammatory response.

The antagonistic peptides used in this study were selected from a phage display library and specifically antagonize extracellular loop 1 and loop 2 of CCR5 [16]. Previously, we found that antagonistic peptides that specifically bind to the second extracellular loop of CCR5 inhibit inflammatory cell infiltration in the lungs of asthmatic mice by downregulating the TNF-*α*/NF-*κ*B pathway [17]. However, the potential of peptides antagonistic to CCR5 in the treatment of inflammatory diseases has been investigated only rarely. Therefore, exploration of the relationship between CCR5 and the IL-23/Th17 pathway in asthma is of great practical significance for the targeted treatment of asthma.

## 2. Materials and Methods

### 2.1. Animals

Six- to eight-week-old female BALB/c mice (20-22 g) were purchased from the Laboratory Animal Center of Sun Yat-sen University and housed under specific pathogen-free conditions. All the experiments were approved by the Ethics Committee for Animal Studies at Sun Yat-sen University, China (IACUC-2019-000097).

### 2.2. Preparation for Antagonistic Peptides of CCR5

The protein database was searched for the amino acid sequences of the first and second extracellular loops of CCR5, and these sequences were chemically synthesized. After three to four rounds of screening with Ph.D.™-7 Phage Display Peptide Library, the specific phages were collected and primarily identified by ELISA. The sequences of the peptides displayed on the selected phages were GHWKVWL and HYIDFRW, and both of these yielded positive results in a phage ELISA [16]. Antagonistic peptides that specifically bind to the first and second extracellular loops of CCR5 (GH and HY peptides) were synthesized by GL Biochem Co., Ltd. (Shanghai, China) to a purity of 95%.

### 2.3. Asthma Model and Pharmacological Intervention

The asthmatic model was prepared as described in a previous study [17]. In brief, mice were sensitized intraperitoneally with 50 *μ*g of OVA (grade V, Sigma-Aldrich Chemical, St. Louis, MO, USA), emulsified in 5 mg of aluminum hydroxide (Al(OH)_3_) to a total volume of 100 *μ*L on days 0 and 5, and then challenged with 100 *μ*g of OVA on days 12, 13, 14, 15, and 16. Twenty-four hours after the last OVA challenge, the mice were subjected to further testing. In this study, the mice were randomly divided into seven groups (*n* = 8 mice) as follows: (1) control group—mice were sensitized and challenged with phosphate-buffered saline (PBS); (2) sensitization group—mice were sensitized with OVA and challenged with PBS; (3) model group—mice were sensitized and challenged with OVA; (4) anti-IL-23p19 group—mice were administered 100 ng of anti-IL-23p19 antibodies (no sodium azide) (eBioscience, San Diego, CA, USA) through continuous intravenous injection for 7 days after the asthma model was established; (5) GH peptide therapy group (GH group)—mice were administered 35 mg/kg GH through continuous intravenous injection for 7 days after the asthma model was established; (6) HY peptide treatment group (HY group)—mice were administered 25 mg/kg HY through continuous intravenous injection for 7 days after the asthma model was established; and (7) anti-IL-23p19 antibody and HY peptide treatment group (anti-IL-23p19 with HY group)—mice were administered 100 ng of anti-IL-23p19 through continuous intravenous injection for 7 days and then 25 mg/kg HY for another 7 days after the asthma model was established. The doses of anti-IL-23p19, GH and HY used in this study were determined based on the results of a preliminary experiment, involved the assessment of behavioral changes, a staining analysis of airway inflammation, and the counting of inflammatory cells in the BALF of mice.

### 2.4. Number of Cells in the BALF

Twenty-four hours after the final treatment was administered, the mice were sacrificed, and the BALF was collected by flushing the lungs three times with 0.5 mL of PBS through an intravenous catheter. The total number of cells in the BALF was counted with an automatic cell counter. In addition, the numbers of lymphocytes, eosinophils, and neutrophils were counted by flow cytometry. Specifically, the expression of cell surface markers was assessed using the following fluorescent dye-conjugated mouse antibodies: PE-Cy7-CD45 (eBioscience, San Diego, CA, USA), Alexa 647-F4/80 (BD Biosciences, Sparks, MD, USA), PE-siglecF (BD Biosciences, Sparks, MD), and FITC-Ly6G (eBioscience, San Diego, CA, USA). The data were collected using a FACSCalibur flow cytometer (BD Biosciences, Sparks, MD, USA).

### 2.5. IL-4 in the BALF

The BALF supernatant was collected, and the IL-4 level was detected by ELISA (R&D Systems, USA) according to the manufacturer's protocol.

### 2.6. Evaluation of Inflammation and Mucus Secretion by Hematoxylin and Eosin (HE) and Periodic Acid-Schiff (PAS) Staining

Twenty-four hours after the final challenge with OVA, the mice were sacrificed, and the right lower lung was placed in 4% paraformaldehyde, embedded in paraffin, and cut into 5 *μ*m sections. The sections were stained with HE or PAS and observed under an Eclipse 80i microscope (Nikon, Japan). In addition, the inflammation score was determined according to the degree of inflammatory cell infiltration around the bronchus [18]. The mucus hypersecretion score was determined according to the percentage of the mucus-positive area of the whole bronchus. The criteria were as follows [19]: 0 points—the percentage of the mucus-positive area of the whole bronchus was less than 5%; 1 point—5–25%; 3 points—50–75%; and 4 points—>75%. All slides were examined in a random blinded fashion by 2 independent investigators.

### 2.7. Real-Time PCR for Determining the mRNA Expression Levels of IL-23p19, IL-23R, IL-17A, LTF, and CCR5

The total RNA from the left upper lung tissues was extracted using the TRIzol reagent (Takara Bio Inc., Otsu, Japan) according to a standard protocol. cDNA samples were obtained with PrimeScript RT Master Mix (Takara Bio Inc., Otsu, Japan) and prepared for PCR using TB Green Premix Ex Taq II (Takara Bio Inc., Otsu, Japan). Subsequently, qRT-PCR analyses were performed on a LC480 instrument (Roche, Basel, Switzerland) using the following primers: *β*-actin—forward GATCAAGATCATTGCTCCTCCTG and reverse AGGGTGTAAAA CGCAGCTCA; IL-23p19—forward CAGCAGCTCTCTCGGAATCTC and reverse TGG ATACGGGGCACATTATTTTT; IL-23R—forward AGAGACACTGATTTGTGGGA AAG and reverse GTTCCAGGTGCATGTCATGTT; IL-17A—forward TCAGCGTGT CCAAACACTGAG and reverse CGCCAAGGGAGTTAAAGACTT; LTF—forward TGATGCCATGACTCTTGATGGT and reverse TCTTTGGTCCCGTAGACTTCAG; and CCR5—forward TTTTCAAGGGTCAGTTCCGAC and reverse GGAAGACCATCAT GTTACCCAC. *β*-Actin was used as the internal control, and the reaction procedure was as follows: predenaturation at 95°C for 10 s; 40 cycles of 95°C for 5 s and 60°C for 32 s; 95°C for 1 min; and 60°C for 15 s for the melting curve analysis. In the experiment, the 2^-*ΔΔ*Ct^ method was used to analyze the relative levels of mRNA transcripts.

### 2.8. Western Blotting for Determining the Protein Expression Levels of IL-23p19, IL-23R, IL-17A, LTF, and CCR5

The total proteins from the right upper lung tissues were extracted by RIPA, and the protein concentration was detected using the standard BCA protocol. The proteins were denatured by heat and electrophoresed by sodium dodecyl sulfate polyacrylamide gel electrophoresis (SDS-PAGE). The separated proteins were then transferred to a polyvinylidene fluoride (PVDF) membrane. The membrane was blocked with 5% dry nonfat milk at room temperature for 1.5 hours and incubated with the primary antibody (1 : 1000 dilution) overnight at 4°C. On the next day, the secondary antibody (1 : 5000 dilution) was added, and the membrane was incubated for 1 hour at room temperature and washed with TBST buffer. ECL images were then obtained, and the results were analyzed using the Hyperfilm G:BOX XT4 system (Syngene, Cambridge, UK). GAPDH was used as the reference protein. The IL-23p19, IL-17A, and CCR5 antibodies were purchased from Santa Cruz Biotechnology (Dallas, TA, USA). IL-23R antibody was purchased from Thermo Fisher Scientific (Waltham, MA, USA), and LTF antibody was purchased from Abcam (Cambridge, MA, USA).

### 2.9. Positive Production Rates of Th17 Cells in the Thymus, Spleen, and Peripheral Blood as Determined by Flow Cytometry

The lymphocytes in the thymus, spleen, and peripheral blood were extracted with lymphocyte separation medium and washed with PBS. The obtained mononuclear cells (1 × 10^6^/mL) were incubated with 2 *μ*L of stimulation cocktail (eBioscience, San Diego, CA, USA) in RPMI 1640 with 10% fetal calf serum at 5% CO_2_ and 37°C for 5 hours. After centrifuging and resuspending with PBS, the cells were stained with surface-specific Abs (anti-CD3e-APC and anti-CD4-FITC, all purchased from eBioscience (San Diego, CA, USA)) at 4°C in the dark for 30 min. After centrifuging and resuspending with PBS, the suspension was incubated with fixation/permeabilization solution (BD Biosciences, Sparks, MD, USA) and stained with anti-IL-17A-PE (eBioscience, San Diego, CA, USA) at 4°C in the dark for 30 min. Acquisition was done on a FACSCalibur flow cytometer (BD Biosciences, Sparks, MD, USA), and FlowJo software was used for analysis.

### 2.10. Analysis of the Correlation between CCR5 and IL-23p19

The results from the PCR and western blot analyses of CCR5 and IL-23p19 were obtained, and Pearson's correlation analysis was used to evaluate the relationship between CCR5 and IL-23p19.

### 2.11. Statistical Analysis

SPSS 22.0 and GraphPad Prism 6 software were used for the analysis. The measurement data are expressed as the means ± standard error of the mean (SEM). One-way analysis of variance (ANOVA) was applied for the normal distribution of datasets. If the analysis of variance was significant, the least significant difference (LSD) method was used to compare the groups. Pearson's correlation test was used to analyze the relationships. *P* values less than 0.05 were considered statistically significant.

## 3. Results

### 3.1. Effects of Peptides of CCR5 and Anti-IL-23p19 mAb on the Pathology and Inflammatory Scores of the Mouse Lung Tissue

**Figure 1:**
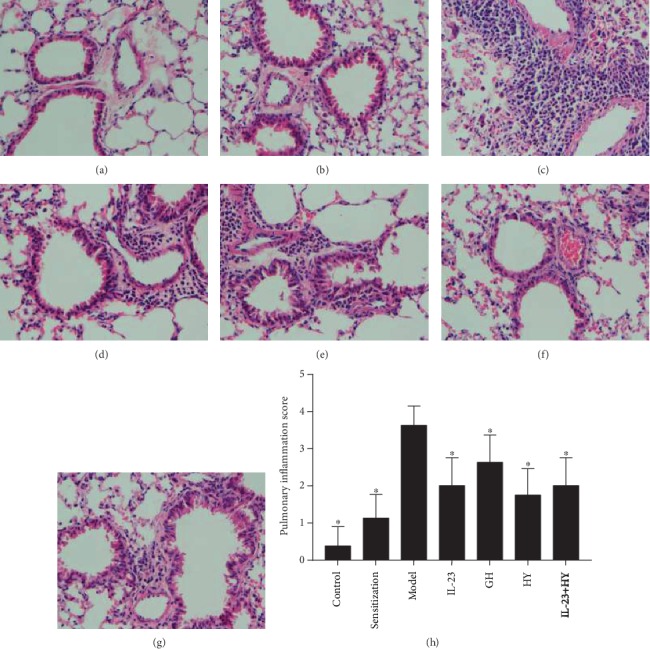
Effects of peptides of CCR5 and anti-IL-23p19 mAb on the airway inflammation. (a)–(g) show photomicrographs of HE staining (original magnification: 400×) of lung sections in the control group, sensitization group, model group, anti-IL-23p19 group, GH group, HY group, and anti-IL-23p19 with the HY group, respectively. (h) Inflammation score of mouse lung tissue in each group. Values are the means ± SD (*n* = 8). ^∗^*P* < 0.05 compared with the model group.

Microscopic observations of the blank control mice revealed no or few inflammatory cells in the airway, no thickening of the airway wall, and no mucus deposition (Figure 1(a)). However, the OVA-sensitized mice exhibited a slightly increased number of inflammatory cells and slight damage to bronchial epithelial cells compared the control mice (Figure 1(b)). Observations of the mice belonging to the model group showed a significant number of inflammatory cells in the peribronchial area, hypersecretion of mucus, and thickening of the bronchus (Figure 1(c)). The administration of anti-IL-23p19 mAb, GH peptide, HY peptide, or IL-23p19 mAb with the HY peptide to the allergic mice significantly reduced the infiltration of inflammatory cells in lung tissue compared with the number of infiltrated cells found in the mice belonging to the model group (Figures 1(d)–1(g)). The degree of airway inflammation was notably increased in the asthmatic group compared with the control group (Figure 1(h)). The inflammation score of the other treatment groups was significantly lower than that of the model group (Figure 1).

### 3.2. Effects of Peptides of CCR5 and Anti-IL-23p19 mAb on Mucus Secretion in the Mice

**Figure 2:**
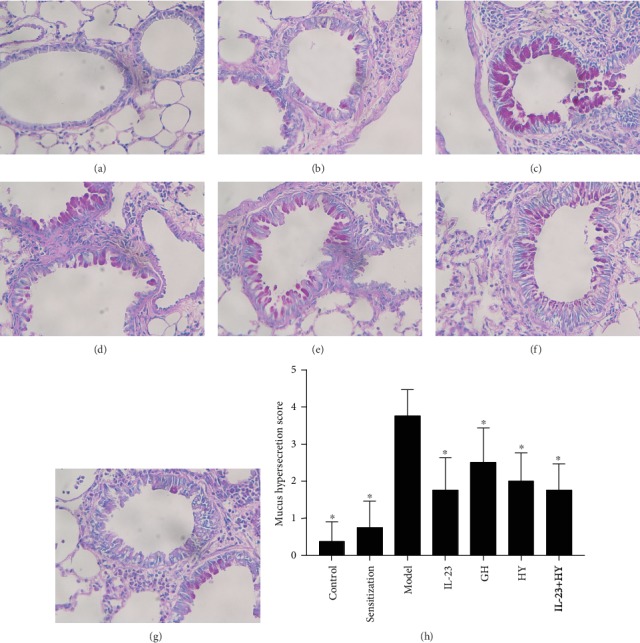
Effects of peptides of CCR5 and anti-IL-23p19 mAb on the airway mucus secretion. (a)–(g) show photomicrographs of PAS staining (original magnification: 400x) of lung sections in the control group, sensitization group, model group, anti-IL-23p19 group, GH group, HY group, and anti-IL-23p19 with the HY group, respectively. (h) Mucus hypersecretion score of mouse lung tissue in each group. Values are the means ± SD (*n* = 8). ^∗^*P* < 0.05 compared with the model group.

As shown in Figure 2(a), no or little mucus secretion was observed. However, the degrees of mucus secretion and goblet cell hyperplasia were slightly increased in the OVA-sensitized mice compared with the control mice (Figure 2(b)). The mice belonging to the model group showed hypersecretion of mucus and thickening of the bronchus (Figure 2(c)). In contrast, the administration of anti-IL-23p19 mAb, GH peptide, HY peptide, or IL-23p19 mAb with HY peptide to the allergic mice alleviated the degrees of mucus hypersecretion and goblet cell hyperplasia (Figures 2(d)–2(g)). The mucus hypersecretion scores obtained for the other treatment groups were significantly lower than those of the model group (Figure 2).

### 3.3. Effects of Peptides of CCR5 and Anti-IL-23p19 mAb on the Differential Cell Counts and Level of IL-4 in the BALF

**Figure 3:**
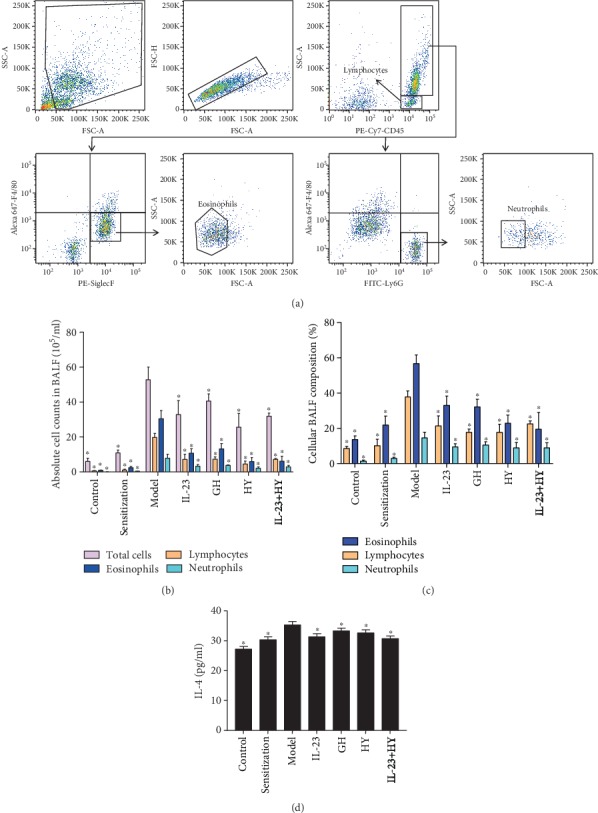
Effects of peptides of CCR5 and anti-IL-23p19 mAb on the inflammatory cell counts and level of IL-4 in the BALF. (a) Flow cytometry gating strategy for inflammatory cell populations in the BALF. Absolute cell counts (b) and cellular composition (c) in the BALF. (d) Quantification of IL-4 in the BALF by means of ELISA. Values are the means ± SD (*n* = 8). ^∗^*P* < 0.05 compared with the model group.

GH peptide, HY peptide, and anti-IL-23p19 mAb could reduce the number of the various types of inflammatory cells and the level of IL-4 in the BALF. The increases in the total inflammatory cells, namely, lymphocytes, eosinophils, and neutrophils, and IL-4 in the BALF from the model group were compared with those in the BALF from the control group (Figure 3). Lymphocytes and eosinophils accounted for the major proportion of the cells in the BALF from both the sensitization and model groups (Figures 3(b) and 3(c)). The total inflammatory cells, the classification of the cells, and the level of IL-4 were decreased in the GH peptide, HY peptide, and anti-IL-23p19 groups compared with those in the model group (Figure 3).

### 3.4. Effects of Peptides of CCR5 and Anti-IL-23p19 mAb on the Expression of IL-23p19, IL-23R, IL-17A, and LTF

**Figure 4:**
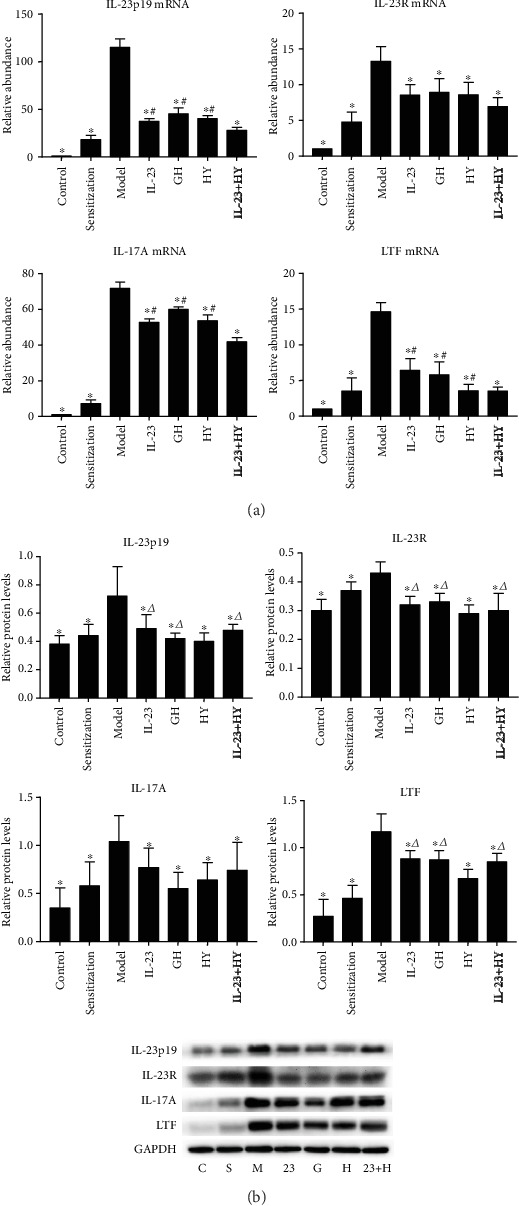
Effects of peptides of CCR5 and anti-IL-23p19 mAb on the expression of IL-23p19, IL-23R, IL-17A, and LTF. (a) IL-23p19, IL-23R, IL-17A, and LTF mRNA levels compared with each other among the 7 groups. (b) Fold change of expression levels of the IL-23p19, IL-23R, IL-17A, and LTF proteins. C: control group; S: sensitization group; M: model group; 23: anti-IL-23p19 group; G: GH group; H: HY group; and 23+H: anti-IL-23p19 with the HY group. Values are the means ± SD (*n* = 8). ^∗^*P* < 0.05 compared with the model group. ^#^*P* < 0.05 compared with the anti-IL-23p19+HY group. ^△^*P* < 0.05 compared with the HY group.

The IL-23p19, IL-23R, IL-17A, and LTF mRNA and protein expression levels were increased by approximately 115.15-fold, 13.24-fold, 71.81-fold, and 14.63-fold and 1.89-fold, 1.43-fold, 2.97-fold, and 4.33-fold, respectively, in the model group compared with the control group (Figure 4). The administration of anti-IL-23p19 mAb, GH, HY, or anti-IL-23p19 mAb with HY significantly decreased the expression of IL-23p19, IL-23R, IL-17A, and LTF in the drug intervention groups compared with those in the model group (Figure 4), and the IL-23p19, IL-17A, and LTF mRNA expression levels in the anti-IL-23p19 with HY peptide group were significantly decreased compared to the levels found in the other three treatment groups (Figure 4(a)). The administration of the HY peptide to the model group induced the most obvious decrease in the IL-23p19, IL-23R, and LTF protein expression levels among the four treatment groups (Figure 4(b)).

### 3.5. Effects of Peptides of CCR5 and Anti-IL-23p19 mAb on the Number of Th17 Cells Positively Produced in Asthmatic Mice

**Figure 5:**
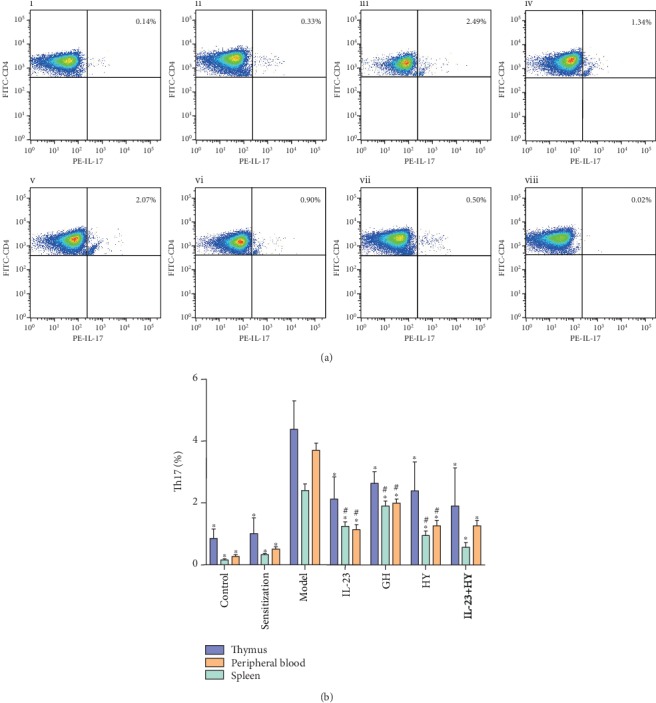
Effects of peptides of CCR5 and anti-IL-23p19 mAb on the number of Th17 cells positively produced. (a) Representative results of the percentage of the Th17 cells in the CD3^+^CD4^+^ gate of the spleen compared the (i) control, (ii) sensitization, (iii) model, (iv) anti-IL-23p19, (v) GH, (vi) HY, and (vii) anti-IL-23p19 with HY groups, as analyzed by using flow cytometry. (vii) The FMO control of IL-17 staining. (b) Histogram showing the proportion of Th17 cells in the thymus, spleen, and peripheral blood. Values are the means ± SD (*n* = 8). ^∗^*P* < 0.05 compared with the model group. ^#^*P* < 0.05 compared with the anti-IL-23p19+HY group.

The numbers of IL-17-expressing Th17 cells in the thymus, spleen, and peripheral blood were increased in the allergic group compared the control group (Figure 5). In addition, the administration of anti-IL-23p19 mAb, GH, HY, or anti-IL-23p19 with HY significantly downregulated the positive production rate of Th17 cells in the thymus, spleen, and peripheral blood of the mice compared with the model group. Strikingly, treatment with the anti-IL-23p19 with HY peptide led to the most substantial decrease in the positive production rate of Th17 cells among the four treatment groups (Figure 5).

### 3.6. Effects of Peptides of CCR5 and Anti-IL-23p19 mAb on the Relationship between CCR5 and IL-23p19 in Asthmatic Mice

**Figure 6:**
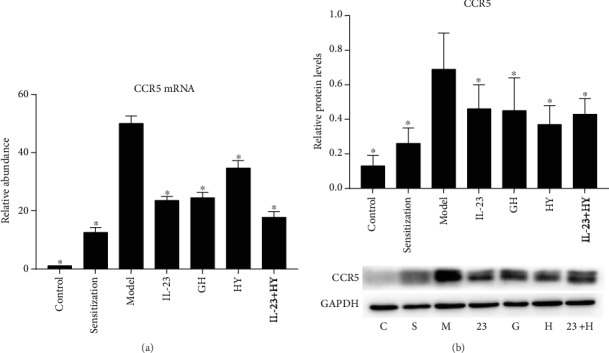
Effects of peptides of CCR5 and anti-IL-23p19 mAb on the relationship between CCR5 and IL-23p19. CCR5 mRNA was analyzed by PCR (a), and CCR5 protein was analyzed by western blotting (b) after the administration of the peptides and/or anti-IL-23p19 to mice. C: control group; S: sensitization group; M: model group; 23: anti-IL-23p19 group; G: GH group; H: HY group; 23+H: anti-IL-23p19 with HY group. Values are the means ± SD (*n* = 8). ^∗^*P* < 0.05 compared with the model group.

Both the mRNA and protein levels of CCR5 were increased in the lung tissues after OVA exposure. The expression of CCR5 decreased after the administration of anti-IL-23p19 mAb, GH, HY, or anti-IL-23p19 mAb with HY (Figures 6(a) and 6(b)). However, the difference among the four treatment groups was not obvious. The protein expression levels of CCR5 and IL-23p19 were significantly and positively correlated in the GH group, HY group, and anti-IL-23p19 with the HY group but not in the anti-IL-23p19 group, and the correlation coefficients in the GH, HY, and IL-23p19 with HY groups were 0.790, 0.747, and 0.799, respectively (Table 1).

## 4. Discussion

The immune network that regulates asthma is very complex. Studies have shown that the IL-23/Th17 signaling pathway is essential for the occurrence and development of the immune response in asthma [20]. This pathway is mainly activated by IL-23, which is the product of antigen-presenting cells such as DCs and macrophages. The release of IL-23 accompanied by the release of transforming growth factor *β* (TGF-*β*), IL-1*β*, IL-21, and IL-6 promotes the activation of naïve CD4^+^ T cells. These proinflammatory cytokines mainly stimulate retinoid-related orphan nuclear receptor *γ*t (ROR*γ*t) and signal transducer and activator of transcription 3 (STAT3), leading to the differentiation of Th17 cells. Subsequently, the Th17 cells secrete IL-17A, IL-17F, IL-22, and other inflammatory cytokines that participate in the immune response [21].

The main pathological features of patients with severe asthma are increases in IL-17 inflammatory cytokines and neutrophil infiltration in the airway, and patients with Th17-mediated neutrophilic asthma are often unresponsive to corticosteroid treatment [22]. The in vivo experiments confirmed that IL-23 and Th17 cells are involved not only in the recruitment of neutrophils to the airway but also in the enhancement of Th2-mediated eosinophilic inflammation [11]. In addition, IL-23 plays an important role in the upregulation of the allergic response in an asthmatic mouse model and the changes in the levels of IL-17 and the numbers of Tc17 and Th17 cells in the mice induced by treatment with anti-IL-23 mAb [23].

In this study, the results showed that the sensitization and challenge of OVA resulted in high levels of inflammatory cell infiltration, including lymphocytes, eosinophils, and neutrophils, mucus hypersecretion, and higher expression of IL-4 in the mouse lung tissue and BALF. Some studies have suggested that OVA induces inflammatory cell recruitment, mucus hypersecretion, and airway hyperresponsiveness in mice [24]. Moreover, our results showed that the administration of anti-IL-23p19 mAb decreased the levels of IL-23p19 and IL-17A in the airway and reduced the recruitment of inflammatory cells in the BALF, and these findings are consistent with those from the above-described studies. Therefore, an acute asthma model induced by OVA was used in this study, and the anti-IL-23p19 group served as the positive control group for evaluating the effect of peptides antagonistic to CCR5 on airway inflammation in a murine model of asthma.

CCR5 is a type of CC chemokine receptor that belongs to the G protein-coupled receptor family and consists of three extracellular loops and three intracellular loops [25]. As reported, bone marrow-derived lymphocytes differentiate into mature functional CD4^+^ T cells in the thymus and then migrate to secondary lymphoid organs such as the spleen and lymph nodes. During inflammation, antigen-presenting cells, such as DCs, macrophages, and monocytes, express a large number of chemokine receptors and subsequently migrate to the spleen. Cytokines secreted by these antigen-presenting cells function in the differentiation of naïve T cells, which can pass through the blood vessel wall to reach inflamed tissues [26]. CCR5 plays an important role in the migration of and cytokine secretion from antigen-presenting cells [27, 28]. Decreased airway hyperresponsiveness and reduced inflammatory cell infiltration can be observed in CCR5^−/−^ mice [29]. In addition, it has been shown that DAPTA (a type of CCR5 antagonistic peptide) can effectively downregulate immune responses by reducing the expression of CCR5, IL-17A, and other cytokines [30]. In addition, in a previous study, we found that the administration of an antagonistic peptide that specifically binds to the second extracellular loop of CCR5 alleviates the pulmonary inflammatory response in asthmatic mice by reducing the expression of the proinflammatory factor TNF-*α* [17]. Therefore, the suppression of proinflammatory cytokines based on binding of CCR5 with its antagonist may be an attractive strategy for the treatment of asthma and potentially other inflammatory diseases.

In the present study, treatment with GH or HY significantly reduced the number of inflammatory cells in a mouse asthmatic model, reduced the damage to the airway epithelium and the secretion of mucus, and decreased the number of lymphocytes, eosinophils, and neutrophils in the BALF. In addition, the administration of GH, HY, or anti-IL-23p19 mAb decreased the levels of IL-23/Th17 pathway-related cytokines (IL-23p19, IL-23R, and IL-17A) and LTF, which are mainly produced by neutrophils in the lung tissue, and this finding suggests that GH, HY and anti-IL-23p19 mAb exert inhibitory effects on the IL-23/Th17 pathway. To further analyze the effects and mechanism of GH or HY on this pathway, we shifted our focus to the distribution of Th17. The results showed that the positive rate of Th17 production was significantly decreased not only in the thymus but also in the spleen and peripheral blood of asthmatic mice after treatment with GH, HY, or anti-IL-23p19 mAb. This finding suggested that treatment with antagonistic peptides and/or anti-IL-23p19 mAb reduced the level of IL-17A in the lungs by decreasing the amount of Th17 cells in the lymphatic circulation. However, the exact mechanism through which the antagonistic peptide of CCR5 reduces the Th17 cell population remains to be explored. The results showed that the production of CCR5 in the asthmatic response was reduced after treatment with GH, HY, and anti-IL-23p19 mAb, which suggests that antagonistic peptides and anti-IL-23p19 mAb directly or indirectly inhibit the expression of CCR5. Furthermore, Pearson's correlation analysis of the CCR5 and IL-23p19 protein expression levels showed a significantly positive correlation in the GH, HY, and anti-IL-23p19 with HY groups but not in the anti-IL-23p19 group, which suggests that CCR5 is involved in the regulation of the IL-23/Th17 pathway. In addition, some studies have shown that DCs play a major role in the pathogenesis of asthma because IL-23, which is a Th17-induction factor, is usually produced by DCs and macrophages, and is sufficient to induce IL-17A production in naïve CD4^+^ T cells [31]. Therefore, the modulation of DC function by CCR5 inhibition, including IL-23 production, may be the mechanism through which the majority of Th17 is suppressed by the GH or HY peptide. Our study also demonstrated that administration of the GH or HY peptide might reduce the inflammatory response in asthmatic mice by inhibiting the ability of CCR5 to regulate the IL-23/Th17 pathway.

This study found a significant difference in the levels of IL-23/Th17 pathway-related proteins (IL-23p19, IL-23R, and LTF) after administration of the GH or HY peptide, which revealed that the HY peptide exerts a stronger downregulatory effect on inflammation compared with the effect of the GH peptide, but significant differences were not observed in the mRNA levels. A previous study showed that the natural ligands of CCR5, such as RANTES, MIP-1a, and MIP-1*β*, exert specific biological effects by binding to the N terminus or the second extracellular loop of CCR5 [32, 33]. Indeed, a larger sample size is needed to obtain specific results. In addition, the HY peptide exhibited a greater effect on the downregulation of the inflammatory response compared with the effect of anti-IL-23p19 in the levels of pathway-related proteins, and this finding suggests that the antagonistic peptides of CCR5 exert a powerful anti-inflammatory effect on inflammatory airway disease. Currently, a good therapeutic effect has been observed in cases of psoriasis and Crohn's disease treated by targeting IL-23 [34, 35]. This study also provides an experimental basis for the targeted therapy of asthma using antagonistic peptides of CCR5. Specifically, GH and HY are inexpensive, short peptide compounds that exhibit high specificity, high affinity, and high safety, which makes them great prospects for clinical application [16].

In addition, our study found a decrease in the positive production rate of Th17 cells in the spleen and peripheral blood of asthmatic mice, and the effect of anti-IL-23p19 mAb with HY was greater than those obtained with the other treatments. It is worth noting that the anti-IL-23p19 mAb intervention resulted in blockade of the IL-23/Th17 pathway, and the administration of anti-IL-23p19 with HY induced a more pronounced decrease in the Th17 response compared with treatment with the anti-IL-23p19 mAb alone. Therefore, the HY peptide could alleviate inflammation through not only the abovementioned IL-23/Th17 pathway but also other mechanisms that could result in the regulation of inflammation. A previous study conducted by Song et al. [36] showed that GH and HY peptides could inhibit the infiltration of inflammatory cells in rats with colitis by regulating the NF-*κ*B/TNF-*α* signaling pathway. In addition, administration of the HY peptide to the asthmatic mice upregulated the expression levels of autophagy-related genes that inhibit the inflammatory response [37]. These studies further support the explanation that the HY peptide might regulate the immune response through a variety of mechanisms.

## 5. Conclusion

In conclusion, antagonistic peptides that specifically bind to the first and second extracellular loops of CCR5 suppress the development of airway inflammation in asthmatic mice, possibly by inhibiting the CCR5-mediated IL-23/Th17 signaling pathway, and a variety of mechanisms might be responsible for the HY peptide-mediated suppression of airway inflammation. This study also emphasizes that the inhibition of CCR5 in bronchial asthma could be a promising research direction and treatment strategy.

## Figures and Tables

**Table 1:** Correlation between the expression of the CCR5 and IL-23p19 proteins in the treatment groups (*n* = 8).

Treatment groups	*r*	*P* value
Anti-IL-23p19 group	0.612	0.080
GH group	0.790	0.011
HY group	0.747	0.021
Anti-IL-23p19 with HY group	0.799	0.010

## Data Availability

The data used to support the findings of this study are available from the corresponding author upon request.
